# Analysis of Silicon Quantum Dots and Serum Proteins Interactions Using Asymmetrical Flow Field-Flow Fractionation

**DOI:** 10.1021/acs.langmuir.3c00109

**Published:** 2023-05-24

**Authors:** Usharani Nagarajan, Sourov Chandra, Tomohiko Yamazaki, Naoto Shirahata, Françoise M. Winnik

**Affiliations:** † International Center for Materials Nanoarchitectonics (MANA), National Institute for Materials Science (NIMS), 1-1 Namiki, Tsukuba 305-0044, Ibaraki, Japan; ‡ Department of Applied Physics, Aalto University, P.O. Box 15100, FI-00076 Espoo, Aalto, Finland; § Research Center for Functional Materials, National Institute for Materials Science (NIMS), 1-2-1 Sengen, Tsukuba 305-0047, Ibaraki, Japan; ∥ Graduate School of Chemical Sciences and Engineering, Hokkaido University, Kita 13, Nishi 8, Kita-ku, Sapporo 060-0814, Japan; ⊥ Department of Physics, Chuo University, 1-13-27 Kasuga, Bunkyo, Tokyo 112-8551, Japan; # Department of Chemistry, Faculty of Science, University of Helsinki, 00014 Helsinki, Finland

## Abstract

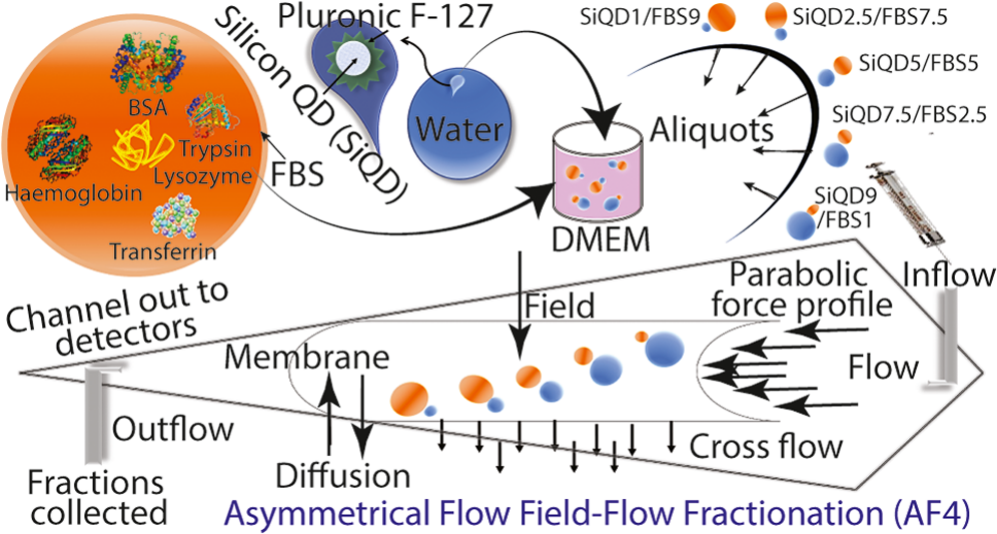

Semiconductor nanocrystals or quantum dots (QDs) have gained significant attention in biomedical research as versatile probes for imaging, sensing, and therapies. However, the interactions between proteins and QDs, which are crucial for their use in biological applications, are not yet fully understood. Asymmetric flow field-flow fractionation (AF4) is a promising method for analyzing the interactions of proteins with QDs. This technique uses a combination of hydrodynamic and centrifugal forces to separate and fractionate particles based on their size and shape. By coupling AF4 with other techniques, such as fluorescence spectroscopy and multi-angle light scattering, it is possible to determine the binding affinity and stoichiometry of protein–QD interactions. Herein, this approach has been utilized to determine the interaction between fetal bovine serum (FBS) and silicon quantum dots (SiQDs). Unlike metal-containing conventional QDs, SiQDs are highly biocompatible and photostable in nature, making them attractive for a wide range of biomedical applications. In this study, AF4 has provided crucial information on the size and shape of the FBS/SiQD complexes, their elution profile, and their interaction with serum components in real time. The differential scanning microcalorimetric technique has also been employed to monitor the thermodynamic behavior of proteins in the presence of SiQDs. We have investigated their binding mechanisms by incubating them at temperatures below and above the protein denaturation. This study yields various significant characteristics such as their hydrodynamic radius, size distribution, and conformational behavior. The compositions of SiQD and FBS influence the size distribution of their bioconjugates; the size increases by intensifying the concentration of FBS, with their hydrodynamic radii ranging between 150 and 300 nm. The results signify that in the alliance of SiQDs to the system, there is an augmentation of the denaturation point of the proteins and hence their thermal stability, providing a more comprehensive understanding of the interactions between FBS and QDs.

## Introduction

Zero-dimensional quantum dots (QDs) are the most promising class of luminescent nanomaterials, significantly useful in electronics, devices, photonics, photocatalysis, as well as biomedical imaging and therapies.^1−4^ Although most of the available QDs are either cytotoxic or suffering from photobleaching,^5,6^ silicon-based quantum dots are highly biocompatible and extremely photostable in nature.^7,8^ Because of their unique optical properties such as tunable emission wavelengths, high photoluminescence (PL) quantum yields, and excellent photostability, near-IR (NIR) emitting silicon quantum dots (SiQDs) are intensively useful for *in vivo* and multiphoton biomedical imaging.^9,10^ An NIR emission probe can easily penetrate the deep tissues and organs without photo damage, as well as minimize the background autofluorescence.^11^ The ability of SiQDs to undergo bioconjugation with specific proteins, peptides, and antibodies could be used to further explore their potential applications in numerous bioanalytical devices.^12^ In our previous studies, we have demonstrated the probable mechanism for the interactions of decyl-coated SiQDs/pluronic-F127 particles with different plasma proteins including albumin, fibrinogen, and transferrin.^13^ We have also investigated the molecular interactions of silicon dots with hemoglobin and thrombin.^14^ Several different techniques, e.g., spectrophotometry, size exclusion chromatography, and gel electrophoresis, have been utilized to evaluate the interactions of QDs and proteins.^15^ However, all of them have certain limitations and are unable to provide the quantitative estimations for the formation of QD–protein complexes.

The interactions of proteins and nanomaterials, commonly known as “corona” over the particle surfaces, are widely useful to understand various pathological and physiological processes, including cellular uptake, communications between cells and biofluids, toxicity, cell adhesion, and inflammations.^16^ Many serum proteins are involved in the formation of complexes with QDs or nanoparticles through different modes of attraction that are headed to the receptors of the plasma membrane.^17^ However, understanding the basic interaction between QDs and the serum protein in biological medium is of great interest. Fetal bovine serum (FBS) is the most widely used serum supplement for the *in vitro* cell culture of eukaryotic cells. The rich variety of proteins in FBS maintains the cultured cells in a medium in which they can survive, grow, and divide. The physiological parameters greatly influence the transitional behavior of proteins in the evaluation system.^18^

The characterization of complexes made by protein and QDs could mimick the *in vivo* systemic circulation, leading to an ardent interest in current research. Though it is a natural process, the toxicological effects of these complexes are determined from their mode of interactions and their structural complexities, which are highly attractive in the pipeline of their clinical trials.^19^ In the present study, SiQDs are chosen as the photoactive particles for evaluating their potential application over *in vivo* systems. Although the dynamic light scattering (DLS) technique is the widely accepted method to characterize the conjugation of QDs with FBS for preclinical formulation development, their carrier behavior on *in vivo* testing is often significantly different from their *in vitro* activity. To attain high-resolution fractionation of complex samples, and access to define the particle size distribution, surface charge, and purity of the QDs-protein complex, asymmetrical flow field-flow fractionation (AF4) could be useful as a versatile separation technique to analyze the interactions between proteins and QDs or nanoparticles.^20−22^ This method allows for the separation and characterization of the different species present in a sample, including QDs, proteins, and QD–protein complexes, based on their size and surface properties. It is one of the most efficient separation systems for the dissolved particles in the size range of 1 nm to several micrometers and allows this measurement quantitatively.^23^ Herein, we demonstrate the impact of water-dispersible NIR-emitting SiQDs in the cellular environment using FBS as a mediocre to cells, enabling the physicochemical behavior of SiQDs upon binding with FBS in biologically relevant media for its preclinical evaluation.^24^ The combinations of AF4, DLS, and differential scanning microcalorimetry (DSC) enabled us to determine the most accurate interfaces of protein–corona complexes with superficial fate and densities. The results indeed will be more significant for further studies relating to biosensors and bioimaging due to their photosensitive characteristics.

## Experimental Section

### Materials

Triethoxysilane (TES), 1-octadecene, mesitylene, and pluronic-F127 were purchased from Sigma Aldrich and used as received. All other chemicals were acquired from Wako Pure Chemical Industries Ltd. (Japan). Dulbecco’s modified Eagle media (DMEM) containing 10% fetal bovine serum (FBS) and 1% penicillin–streptomycin were obtained from Gibco (Life Technologies, Carlsbad, California). Ultrapure Milli-Q water (18 Ω) was produced using a Sartorius (Arium 611 UV) water purification system and used in our experiments.

### Preparation of Water-Dispersible Silicon Quantum Dots

NIR-emitting water-dispersible SiQDs were prepared according to our previous method.^7^ Briefly, disproportionation of the hydrolysis product (HSiO_1.5_) of triethoxysilane (TES) yields oxide-embedded SiQDs. Hydride-terminated SiQDs (SiQD-H) were obtained by removal of the oxide matrix with alcoholic hydrofluoric acid. After that, octadecyl-capped SiQDs (SiQD-OD) were prepared via hydrosilylation reaction of hydride-terminated SiQDs with 1-octadecene at 130 °C in mesitylene. SiQD-OD was then isolated by centrifugation at 15 000 rpm for several times with the introduction of a mixture of toluene and methanol (1:1). The transfer of SiQD-OD from toluene into water was done using pluronic-F127 by taking advantage of the high affinity of the poly (propylene oxide) block of F127 for the hydrophobic OD shell over the dots. The sample was emulsified by vortexing for a few minutes and kept at room temperature for 12 h. Subsequently, the toluene was evaporated. While SiQD-OD are highly dispersible in toluene, the OD/Pluronic-F127 double-layer silicon dots (SiQD-OD-P) turn out to be well soluble in water. The resulting aqueous phase was transparent and showed a strong PL, indicating the successful transfer of SiQD-OD into water.

### Particle Characterization

Fourier transform infrared spectroscopy (FTIR) was conducted using a JASCO FTIR 4100 spectrometer, with powder samples placed in the sample holder. UV–visible optical absorption was performed using a JASCO V-650 spectrophotometer. PL and PL excitation (PLE) spectra were recorded using a NanoLog Horiba Jovin Yvon spectrofluorometer with an InGaAs detector for NIR (Hamamatsu Photonics Co., Ltd, Japan). The X-ray diffraction pattern was recorded with a Rigaku Smart lab X-ray diffractometer. High-resolution transmission electron microscopy (HR-TEM) was accomplished using a JEOL JEM 2010, operating at an acceleration voltage of 200 kV.^25^ Samples for HR-TEM analysis were drop-cast from the dilute dispersions of SiQD-OD and SiQD-OD-P in toluene and water, respectively, on ultrathin carbon (<10 nm thickness)-coated copper grid. Absolute PL quantum yield was measured using a C9920-03G system, equipped with a 150 W xenon lamp produced by Hamamatsu Photonics Co., Ltd., Japan. Dilute solutions having absorption in between 0.1 and 0.2 were inserted into the instrument with a 1 cm^2^ quartz cuvette.^26^

### Preparation of the SiQD-OD-P/FBS Complex in Culture Medium

The stock solution of SiQD-OD-P in water and FBS in DMEM of 10% stock solution were used to prepare SiQD-OD-P/FBS bioconjugates of different compositions. Various bioconjugates were prepared by mixing appropriate amounts of stock solutions as given in Table 1. The concentration ratio between SiQD-OD-P and FBS was fixed based on the stable dispersion system. To understand the effect of temperature and time, the solutions were incubated at 25 and 60 °C for about 0 and 24 h, respectively. Subsequently, these samples were used for further characterization.^27^

**Table 1:** Composition of SiQD-OD-P and FBS Bioconjugates in the Biological Medium

	SiQD-OD-P in water		FBS in DMEM	
samples	spent volume (μL)	total amount in spent volume (mg)	spent volume (μL)	total amount in spent volume (mg)
FBS			1000	10
SiQD-OD-P	1000	10		
SiQD9/FBS1	900	9	100	1
SiQD7.5/FBS2.5	750	7.5	250	2.5
SiQD5/FBS5	500	5	500	5
SiQD2.5/FBS7.5	250	2.5	750	7.5
SiQD1/FBS9	100	1	900	9

### Dynamic Light Scattering

A dynamic light scattering instrument (Beckman Coulter Delsa Nano) was used to measure the hydrodynamic diameter *D*_h_ of the SiQD and FBS mixed solutions in batch mode.^28^

### AF4 Instrumentation

The AF4 instrument consists of an isocratic pump (1260 series (G1310B), Agilent Technologies, Santa Clara, CA) attached to a high-performance liquid chromatography (HPLC) manual injection valve (Wyatt Technology, high-performance injection system) with a 20 μL stainless steel sample loop, field/flow control module, and AF4 separation channel (Eclipse, Wyatt Technology, Santa Barbara, CA) with the ceramic frit overlaid by the permeable membrane, multi-angle light scattering (MALS) detector (DAWN 8+, Wyatt Technology), and ultraviolet–visible (UV–vis) absorbance diode array detector (1260 DAD (G1315D), Agilent Technologies).^21^

### AF4 Separation

The AF4 analysis was carried out using the eluent [deionized water (18.2 mΩ) with sodium azide (0.02%)], which was filtered through a 0.1 μm Whatman filter and sonicated before use. The analysis was carried out with the programmed conditions, which included a focusing/injection step of 2 min with a focusing-flow rate of 0.25 mL min^–1^ and an injection flow rate of 0.2 mL min^–1^. This was followed by a focusing/relaxation step of an additional 2 min. About 21.5 μL of the sample was injected into the system. The sample was injected to the channel, with the axial flow and the focus flow opposing each other and concentrating the sample into a small area on the regenerated cellulose membrane (*M*_w_ cutoff 5 kD). The parameters based on focusing, injection, and elution steps are described in Table 2. An elution step was performed with a linear gradient cross-flow rate, with an initial equilibrium of 0.25 mL min^–1^ for about 10 min and then gradually reaching zero in another 10 min. The detector flow rate was maintained as 0.5 mL min^–1^ throughout the analysis. Data collection and analysis were done using ASTRA software (version 5.3.4.15, Wyatt Technology).^29^

**Table 2:** Flow Details of the Method for SiQD/FBS Bioconjugates

channel parameters	membrane	regenerated cellulose (RC)
	molecular weight cutoff membrane	5 kD
	spacer	350 μm
fractionation time	focusing time	1 min
	focusing + injection time	2 min
	elution time	20 min
flow rate and volume, fractionation step	injection volume	21.5 μL
	injection flow rate	0.2 mL min^–1^
	channel flow (*V*_c_) rate	0.5 mL min^–1^
	cross-flow (*V*_x_) rate	0–0.25 mL min^–1^
eluent	water (18.2 mΩ) + NaN_3_ (0.02% w/w)	

### Fraction Collection and Analysis

An aqueous solution of 21.5 μL of SiQD/FBS was injected into the AF4 channel. The two fractions each at F1 (*t* = 18–24 min) and F2 (*t* = 24–30 min) were collected as a pool from repeated injections (∼10 times).^15^ These fractions were dialyzed against distilled water for about 12 h and further lyophilized. These fractions were used for the UV measurements using a UV–VIS–NIR spectrophotometer (JASCO V-570) to measure the absorption of QD and FBS in the fractions and the concentration mixtures were measured using AF4-UV fractogram.^30^

### Differential Scanning Calorimetry

DSC measurements were performed using the Hitachi HT-Seiko instrument SII Exster X DSC 7000 with a cell volume of 20 mg and under an external pressure of about 180 kPa. The heating rate was set at 2 °C min^–1^ in the range of 20–100 °C. The experimental data were analyzed using the DSC analysis software supplied by the manufacturer. Solutions were kept at 25 and 60 °C for 1 h and degassed at 20 °C under mild vacuum for 15 min prior to loading into sample and reference cells.^16^ The reference cell was filled with 20 mg of sapphire (standard). A scan recorded with empty pan in the sample and reference cells was subtracted from the sample data to remove baseline contributions.^31^

## Results and Discussion

### Synthesis and Characterizations

**Figure 1:**
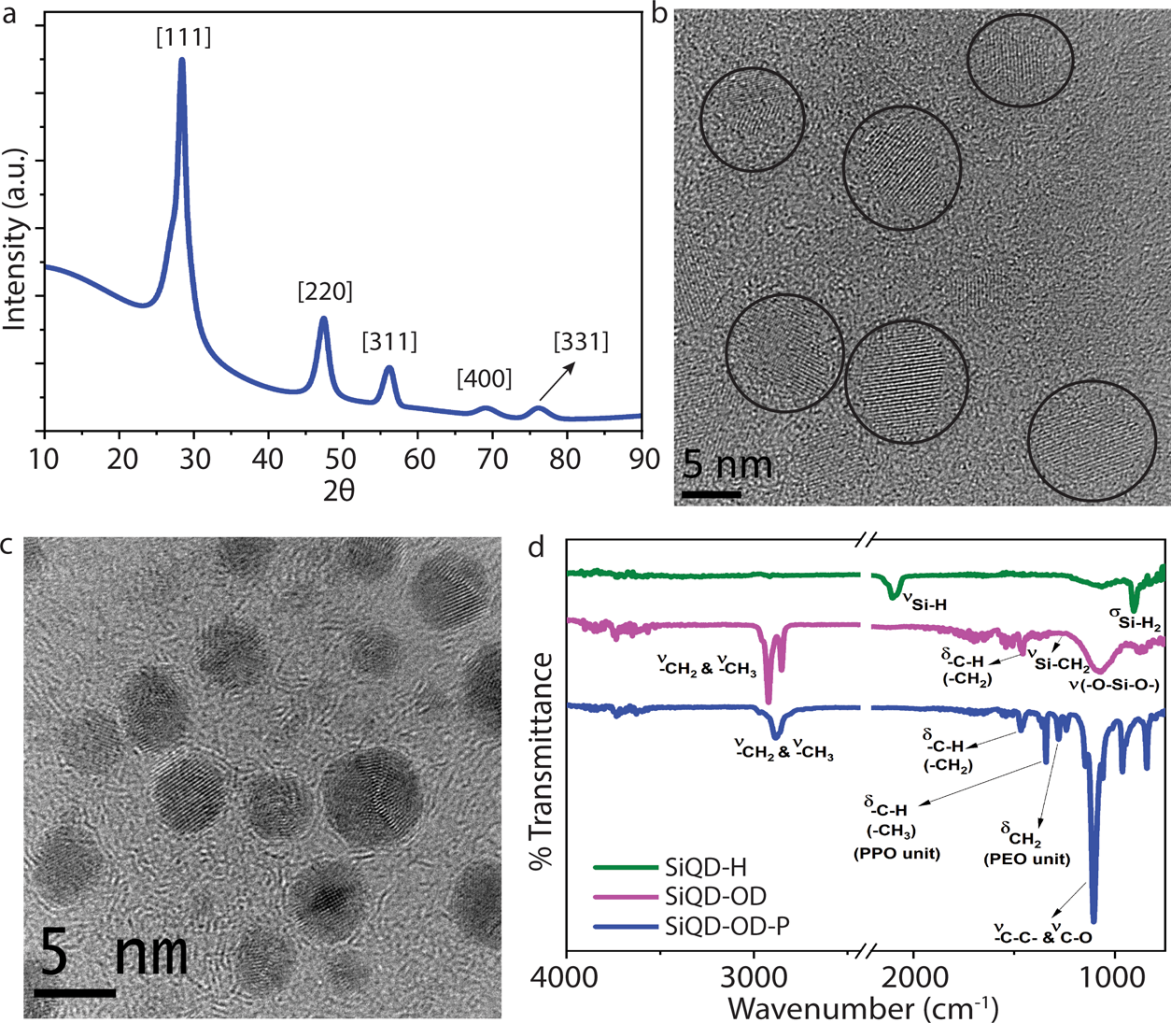
(a) X-ray diffraction pattern of SiQD-OD. HR-TEM image of (b) SiQD-OD and (c) SiQD-OD-P. (d) FTIR spectra of SiQD-H, SiQD-OD, and SiQD-OD-P.

Water-dispersible SiQDs were synthesized according to our previous method.^7^ The X-ray diffraction pattern shown in Figure 1a demonstrates the [111], [220], [311], [400], and [331] planes of the diamond cubic lattice structure of silicon, cited by the peaks at 2θ = 28, 47, 56, 69, and 76, respectively.^32^Figure 1b,c displays the high-resolution TEM images of SiQD-OD and SiQD-OD-P, respectively, indicating that they are well crystalline in nature with a diameter in between 2 and 5 nm. The FTIR spectra shown in Figure 1d illustrate the existence of C–C and C–O bonds (1108 cm^–1^) of the pluronic-F127 counterpart over the SiQD-OD-P. A band at 1343 cm^–1^ is associated with the C–H bending vibration of the −CH_3_ group, whereas the signals at 1285 and 1243 cm^–1^ confirm the existence of −CH_2_ groups of poly(ethylene oxide) blocks (PEO unit) in pluronic-F127.

### Optical Properties

**Figure 2:**
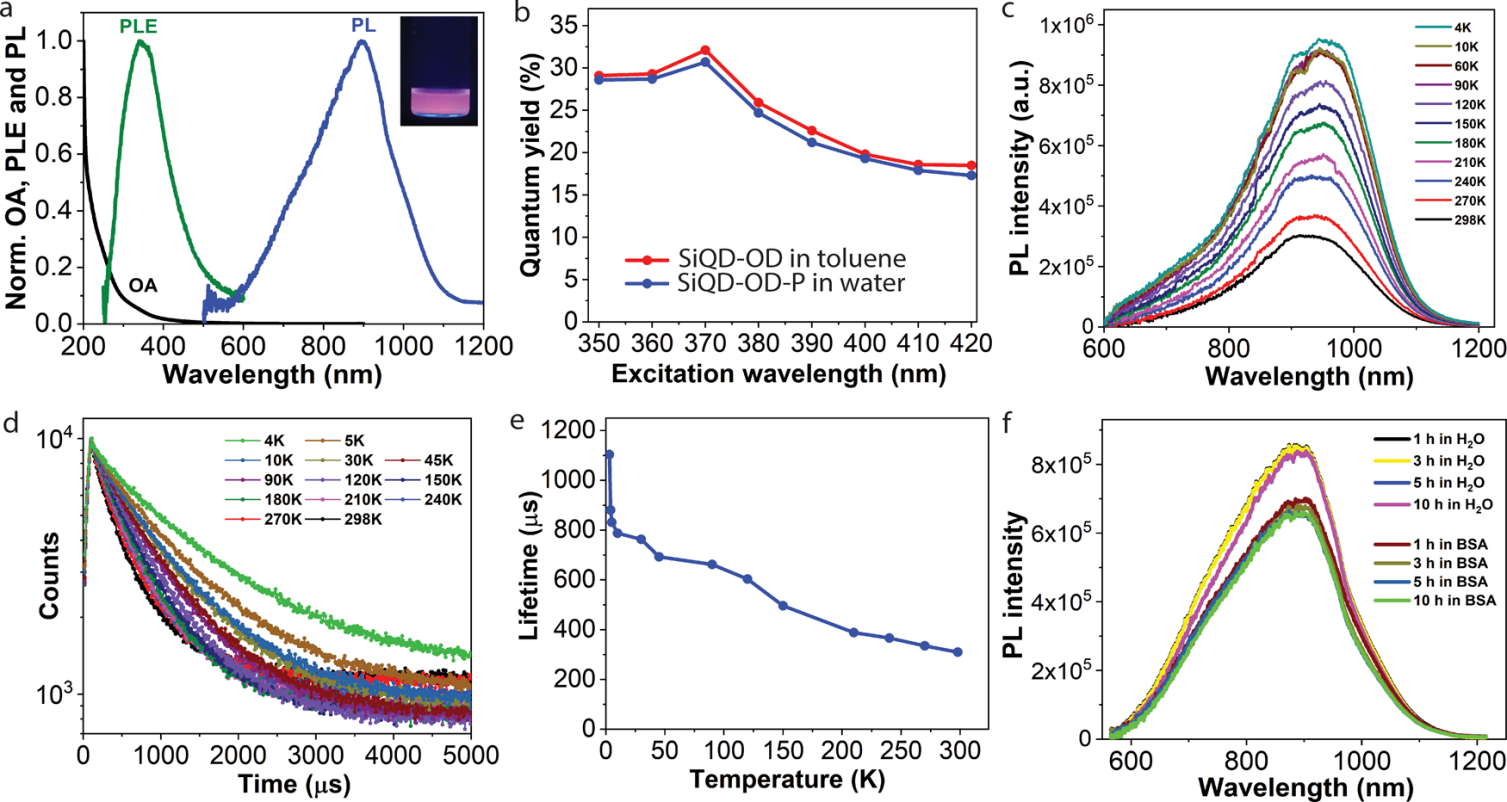
(a) Normalized OA, and steady-state PL (λ_ex_ = 400 nm) and PLE (λ_em_ = 900 nm) spectra of SiQD-OD-P in water. The inset shows the aqueous dispersion of SiQD-OD-P under UV light. (b) PL quantum yields as a function of the excitation wavelength. (c) PL spectra as a function of temperature in the range of 4–298 K for SiQD-OD-P. (d) PL decay profiles as a function of temperature in the range of 4–298 K for SiQD-OD-P. (e) Corresponding plot of temperature vs PL lifetimes and (f) photostability of SiQD-OD-P in water and BSA under continuous UV irradiation for 10 h.

The optical absorbance (OA), photoluminescence (PL) emission, and PL excitation (PLE) spectra of the SiQD-OD-P in water are shown in Figure 2a. While the UV–Vis spectrum clearly reveals a featureless absorption profile with an absorption edge at 350 nm, its corresponding PLE spectrum illustrates a sharp peak at 345 nm. Upon excitation at 350 nm, an intense broad PL spectrum has been observed with the peak maxima at around 900 nm. The particles show strong emissions with high quantum yields (QYs) throughout the excitations in between 350 and 420 nm (Figure 2b). The highest QY has been recorded upon excitation at 370 nm for both SiQD-OD (QY = 32% in toluene) and SiQD-OD-P (QY = 31% in water). So, the PL efficacy of the silicon dots remains almost unaltered upon pluronic-F127 encapsulation. To understand the carrier relaxation and recombination, the PL emission and decay have also been explored as a function of temperature. Figure 2c demonstrates the temperature-dependent PL spectra at 400 nm excitation in the range of 4–298 K. As expected, the PL spectrum of SiQD-OD-P displays a continuous red shift on increasing the PL intensity and broadening upon cooling from 298 to 4 K.^25^Figure 2d,e shows the emission decays at the PL maxima as a function of temperature in the range of 4–298 K. All of the decay profiles are biexponential irrespective of the temperature, demonstrating a fast decay component impending from their surface-trap states along with a slow decay probably due to the radiative recombination of the photogenerated carriers across the band gap. SiQD-OD-P in both aqueous solution and in the presence of serum protein (BSA) is highly photostable in nature; no photobleaching has been observed under continuous UV irradiation (12 W) up to 10 h of our observation (Figure 2f).

To assess the effect of FBS on the optical properties of SiQDs, three different compositions of FBS/QDs have been chosen (Table 3). It is clearly evident that there is a continuous blue shift of the PL spectra on increasing the concentration of FBS as well as the incubation time. A similar trend has also been observed for the average PL lifetime; the decay profile gradually falls to lower values on increasing the composition of FBS in these complexes. The results signify that there is a strong interaction between SiQD-OD-P and FBS in the solution.

**Table 3:** PL Emission and Decay Lifetime of the SiQD-OD-P/FBS Complexes in Solution

sample	incubation time (h)	PL maxima at λ_ex_ = 400 nm	average PL lifetime (μs)
SiQD9/FBS1	0	915 nm	369
	24	901 nm	331
SiQD5/FBS5	0	908 nm	347
	24	878 nm	266
SiQD1/FBS9	0	904 nm	286
	24	870 nm	246

### AF4 Measurements

AF4 operating conditions are optimized based on the native QD elution rate (cross-flow rate = 0.25 mL min^–1^; elution time = 20 min). The hydrodynamic size of the individual SiQD-OD-P in water has been analyzed and found to be in the range of 20–50 nm. Three sample sets were used for the analysis based on dispersion stability, time, and temperature. As given in Table 1, the first sample sets include the individual FBS and SiQD-OD-P along with the different combinations of SiQD-OD-P and FBS in the ratio 9:1, 7.5:2.5, 5:5, 2.5:7.5, and 1:9, respectively. All were incubated for 0 h, i.e., were injected immediately after incubation at 25 °C. The second and third sample sets include the same compositions of SiQD-OD-P/FBS complexes incubated for 24 h at 25 and 60 °C, respectively.

**Figure 3:**
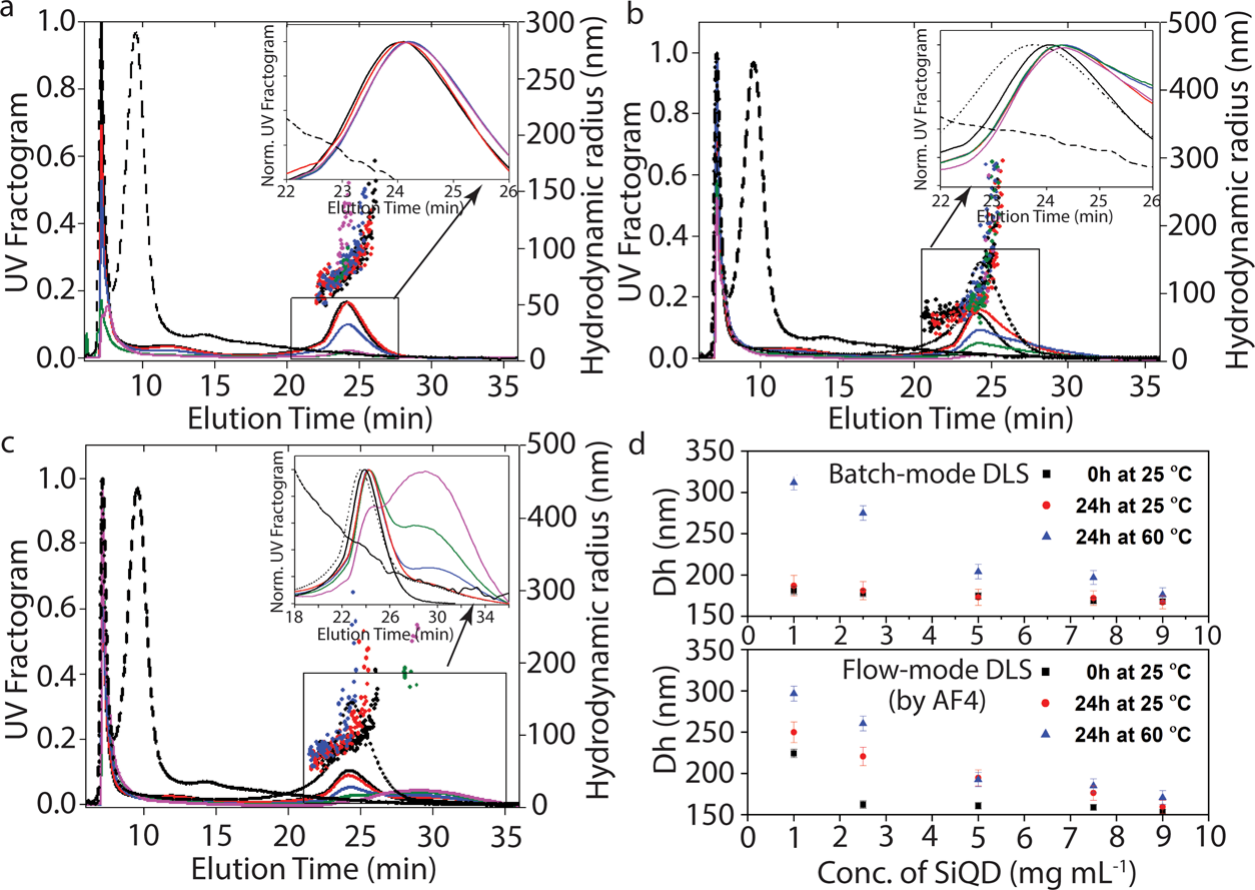
AF4 measurements: UV fractogram (line) and the hydrodynamic radius (scatter) of FBS (black dash), SiQD-OD-P (black dot), and different mixtures of SiQD-OD-P/FBS [SiQD9/FBS1 (black line); SiQD7.5/FBS2.5 (red line); SiQD5/FBS5 (blue line); SiQD2.5/FBS7.5 (green line); SiQD1/FBS9 (magenta line)] incubated for (a) 0 h at 25 °C, (b) 24 h at 25 °C, and (c) 24 h at 60 °C. (d) Hydrodynamic diameter (Dh) of FBS in different samples [SiQD9/FBS1, SiQD7.5/FBS2.5, SiQD5/FBS5, SiQD2.5/FBS7.5, SiQD1/FBS9] as a function of the total amount of SiQD-OD-P using both batch-mode and flow-mode DLS measurements.

In Figure 3, the UV fractogram (line) and the hydrodynamic radius (scatter) of native FBS (black dash) and SiQD-OD-P (black dot) as a function of elution time have been plotted. An increase in the concentration of serum proteins has been represented as different mixtures of SiQD-OD-P/FBS, such as 9:1, 7.5:2.5, 5:5, 2.5:7.5, and 1:9, incubated for 0 h at 25 °C, 24 h at 25 °C, and 24 h at 60 °C, respectively. It is observed that there is a distinct elution peak center at around ∼10 min, which indicates the presence of free FBS in the medium, leaving behind the unbound SiQDs, followed by a void peak at around ∼6 min.^33^ The first set of QD–FBS complexes, which are injected as soon as they interact, exhibit a sigmoidal separation as a function of the elution time, whereas the main peak is centered at 24 min, measuring about 153.4 ± 0.28 nm (Figure 3a). However, the same sample sets which are incubated for about 24 h and monitored for their elution pattern show an increased retention time (Figure 3b). In this case, there is a shift in the peak as a function of the elution time with increase in the retention time. This indicates that the longer the retention time, the more the particle size.^34^ The intensity of the elution peak around 18–30 min seems to be decreasing with decrease in QD concentration. Indeed, it is obvious from the UV fractogram that the elution peak tends to shift to a longer elution time with increase in size compared to both individual SiQD-OD-P and FBS.^32^ This confirms the influence of QDs on FBS to locate the binding motif on them.

For SiQD9/FBS1, the main peak is centered at 26 min, measuring about 162 nm, and keeps increasing with the increase in concentration of FBS to a maximum of about 246 nm (SiQD1/FBS9). This indicates the extent of interaction, and it is shown that the interaction is stable until 24 h of incubation. The SiQDs in turn remain bound to the FBS, illustrating the least chance for aggregation in the separation profile. When SiQDs are allowed to interact with the proteins for more than 48 h, there is an obvious aggregation, which is confirmed by the disrupted elution profile and the formation of turbidity (visually observed, data not shown). A clear reflection of the surface charge plays a crucial role in the system. Finally, the same experimental conditions are employed for the sample sets of different proportions incubated at 60 °C for 24 h (Figure 3c). Eventually, for the SiQD9/FBS1 mixture, the elution peak is centered at 28 min, with a hydrodynamic radius of about 170 nm. In line with the other two systems, the other samples also follow the same behavior as FBS, with a longer elution time of up to 30 min and measuring about 296 nm. This ascertains that excess of FBS increased the absorption behavior with increase in concentration of FBS. The sizes of the SiQD-OD-P/FBS complexes have been determined by batch-mode DLS before AF4 measurement (Figure 3d, upper panel). We observe that the complexes in the three sample sets demonstrate noticeable modifications in size variants based on the extent of their interactions. This shows that there are 2–3-fold increments in size of the complexes in comparison to the native QDs.

All of the sample sets tend to follow a similar trend toward serum proteins with a unique way of separation. Moreover, the recovery of these fractions is estimated, and it is found to be in the range of ∼80% for each injection. Nevertheless, they do not vary much with size, apparently leaving behind the stable particles as well as protein complex in the interaction system. It also permits the quantitative indication of the level of interaction and the relative hydrodynamic radius of the isolated QD–FBS complex and its aggregates.^35^ The fractogram clearly indicates that the serum proteins and the QDs sturdily tether with each other either by hydrogen bonding or through electrostatic interactions based on the carrier medium and the separation background in the AF4 channel.^36^ Moreover, the stability of these particles with respect to incubation time, size determinants, and mass variations substantiates their interaction potency. This is ascertained from the hydrodynamic radius (AF4 separation with flow-mode DLS), as shown in Figure 3d (lower panel). It is evident that with an increase in the FBS concentration there is a narrow increase in size based on the concentration, incubation time, and temperature. This is mainly due to the biomolecular fluctuations of the protein on altering the three-dimensional (3D) structure in the presence of QDs in the biological medium. More precisely, alike proteins, FBS also tends to alter the folding pattern, leading to redundant effects in cellular behavior *in vivo.*(37) Hence, these results obtained from AF4 remain crucial to mimic the tendency of QDs towards cells in *in vitro* conditions.

**Figure 4:**
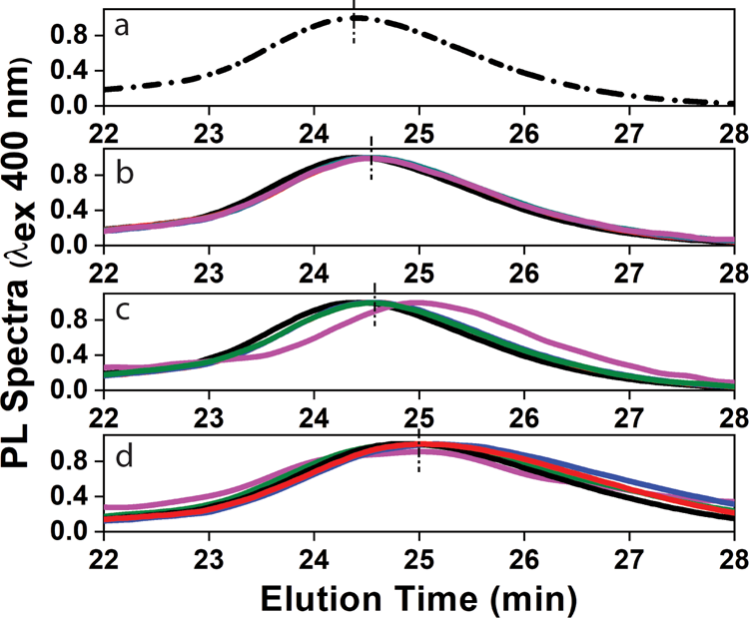
(a) PL spectrum of individual SiQD-OD-P particles using AF4 measurements. PL spectra obtained by AF4 measurements for mixtures of SiQD-OD-P/FBS [SiQD9/FBS1 (black line), SiQD7.5/FBS2.5 (red line), SiQD5/FBS5 (blue line), SiQD2.5/FBS7.5 (green line), and SiQD1/FBS9 (magenta line)] incubated as a function of time and temperature, such as (b) 0 h at 25 °C, (c) 24 h at 25 °C, and (d) 24 h at 60 °C.

The interactions of FBS and QDs have been further confirmed by the effect on the PL spectrum of the native QDs, recorded by means of fluorescence detectors in the AF4 system (Figure 4). We observe a similar trend of the PL fractogram with a slight shift in elution time as a function of incubation time and temperature compared to the individual SiQD-OD-P in water. With increase in the temperature, changes in PL peak position directly indicate the conformational changes in protein. Dzagli et al. have demonstrated that QDs upon interaction with proteins exhibit a linear dependence of the fluorescence peak position on the temperature.^38^ However, in our experiment, FBS/QD mixtures exhibit a more complex behavior that is highly sensitive to structural changes in the protein. The fluorescence fractograms exhibit minor variations in the intensity, maintaining a constant elution pattern like the UV fractograms. This indicates that QDs are stable, with proteins aiding against denaturation. These results indicate the fluorescence behavior of SiQDs annexing FBS, which might be attractive and useful in terms of bioimaging and biosensing applications.^39^

### Calorimetric Measurements for the FBS/SiQD Mixtures

The communications between FBS and SiQDs have also been analyzed by temperature dependence activities, providing an insight to explore the denaturation and conformational changes in the protein. The thermograms of SiQD-OD-P/FBS complexes were recorded upon heating from 20 to 100 °C. The complexes incubated at 25 and 60 °C for about 24 h were studied upon heating using differential scanning calorimetry (data not shown). It is observed that these sample sets present a broad endotherm corresponding to the temperature-induced association of the SiQD-OD-P/FBS mixtures.^40^

**Figure 5:**
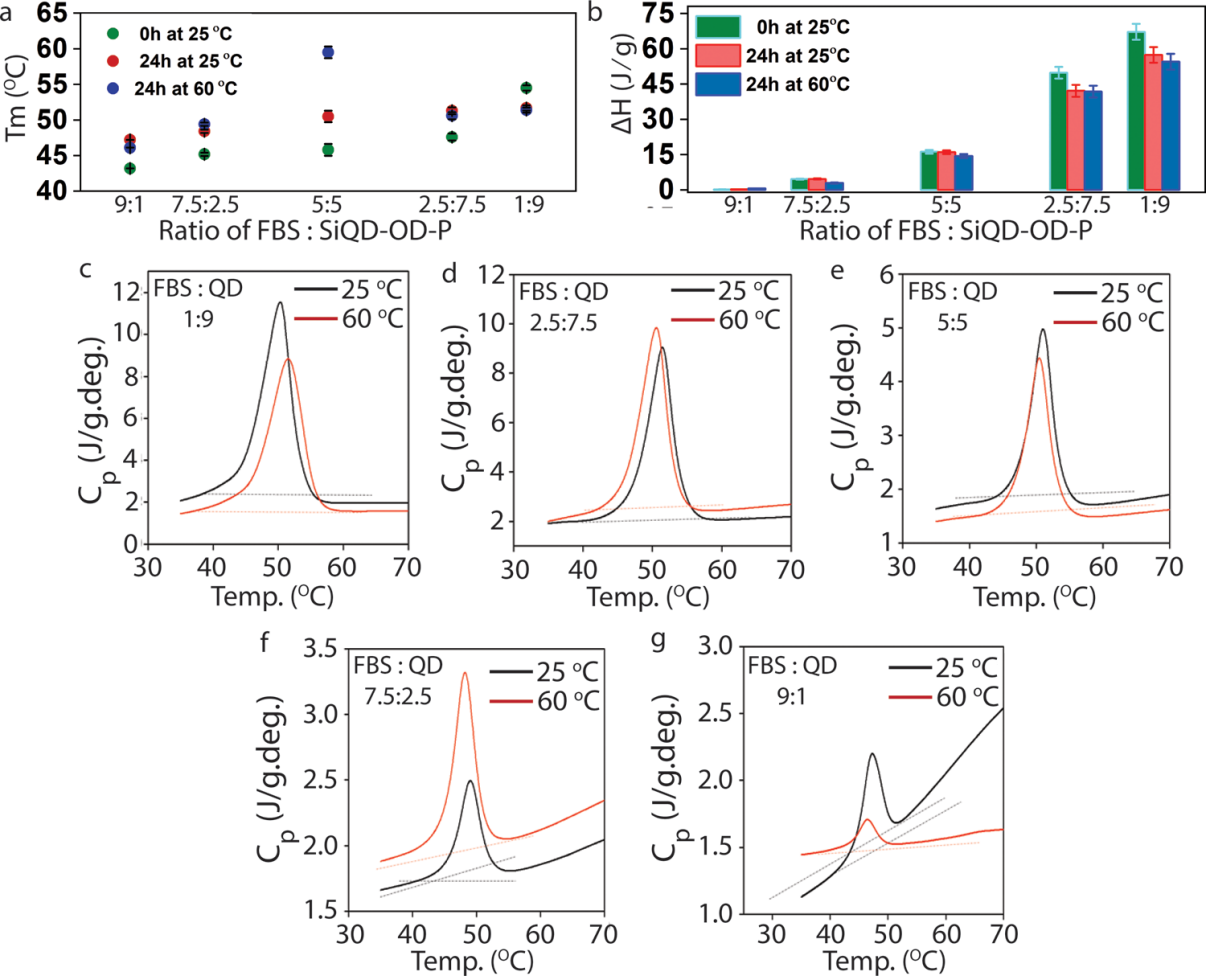
(a) Denaturation temperature (*T*_m_) and (b) thermodynamic parameter (Δ*H*) of the samples on varying the amount of SiQD-OD-P, incubated for 0 h at 25 °C, 24 h at 25 °C, and 24 h at 60 °C. Specific heat capacity of (c) SiQD9/FBS1, (d) SiQD7.5/FBS2.5, (e) SiQD5/FBS5, (f) SiQD2.5/FBS7.5, and (g) SiQD1/FBS9, incubated for 24 h at 25 and 60 °C using differential scanning microcalorimetry (scan rate = 0.2 °C min^–1^).

The denaturation temperature (*T*_m_) seems to be increasing as the concentration of QD increases in the system (Figure 5a). It is observed that the main endotherm peak of the SiQD-OD-P/FBS complex incubated at 25 °C for 0 h is centered at *T*_m_ = 43.2 °C for SiQD1/FBS9 composition, and the *T*_m_ value increases with increase in the ratio of the QDs in this complex. This specifies that SiQD-OD-P plays an important role in stabilizing the structural function of proteins at higher temperatures. Furthermore, for the samples incubated at 25 or 60 °C for 24 h, *T*_m_ seems to be higher than that of the samples without incubations (0 h). In addition, the enthalpy change (Δ*H*) of these samples is rapidly enhanced on increasing the fraction of QDs (Figure 5b), with the concentration measuring about 67.2 mg mL^–1^. Nevertheless, we observe that the structural stability of serum proteins is protected in the biological medium in the presence of QDs.

Moreover, the specific heat capacity (*C*_p_) measurements are carried out for the SiQD-OD-P and FBS sample sets incubated for 24 h at 25 and 60 °C. The calorimetric studies reveal that unfolding of serum proteins involves extensive heat absorption and depends on the concentration of QDs (Figure 5c–g). In the case of FBS, the calorimetric enthalpy has been found to be in good correspondence with the van’t Hoff enthalpy of unfolding. The thermodynamic function indicates protein unfolding/refolding in accordance with the heat capacity of native and unfolded states of the protein over the range of temperature.^41^ It is observed that the baseline for the *C*_p_ vs temperature remains stable for the samples having a higher proportion of QDs. This in turn specifies that the heat capacities of the native and unfolded protein remain parallel at 25 and 60 °C, respectively. With the increase in the concentration of FBS and the time of incubation, the difference in the baselines indicates the changes in the protein folding pattern from its native structure. Upon decrease in the concentration of QD beyond 5:5 FBS/SiQD-OD-P composition, the unfolding of proteins seems to be rapid. Hence, the increasing heat capacity of the native protein with increase in temperature is not simply due to intensifying vibrations of the protein structure but also reflects the accumulation of energy upon heating. These results explain the influence of SiQD-OD-P on the protein’s folding and unfolding patterns.

## Conclusions

The combination of AF4 and DSC has provided valuable insights into the interaction of FBS with SiQDs in biological systems. The unique optical and electronic properties of SiQDs along with high photostability and biocompatibility enable their high-sensitive detection and imaging of biological molecules and processes. The experimental findings have shown that the interaction between FBS and SiQDs is complex and influenced by a variety of factors such as the concentration of particles, proteins, temperature, and incubation time. In this study, the conjugations of FBS and SiQDs have been monitored to mimic the real-time interaction system. The promising results obtained in our investigations suggest that a single AF4 technique has the potentiality to revolutionize the way we study and manipulate biological systems at the nanoscale. It is evident that an increase in size of the FBS/SiQD complex has shifted the elution peak to a longer wavelength in comparison to their native forms. The significant part of this analysis is the extent of aggregate formation and its quantitative determination. The highlights of the findings obtained from AF4 fractograms have clearly indicated (i) the presence of the individual QDs and the complexes and (ii) the distribution or evolution of a homogeneous size range and shape, and (iii) helped in finally evaluating the targeted multicriteria characterizations, including the stability of these complexes in a biological medium. Overall, the structural stabilities and folding patterns of serum proteins in the presence of SiQDs using AF4 and DSC pave the way to future research and development in the field of nanobiotechnology, with the potential to lead to new and innovative approaches for the diagnosis and treatment of various diseases.
